# Incidence of pain flare following palliative radiotherapy for symptomatic bone metastases: multicenter prospective observational study

**DOI:** 10.1186/s12904-015-0045-8

**Published:** 2015-10-01

**Authors:** Alfonso Gomez-Iturriaga, Jon Cacicedo, Arturo Navarro, Virginia Morillo, Patricia Willisch, Claudia Carvajal, Eduardo Hortelano, Jose Luis Lopez-Guerra, Ana Illescas, Francisco Casquero, Olga Del Hoyo, Raquel Ciervide, Ana Irasarri, Jose Ignacio Pijoan, Pedro Bilbao

**Affiliations:** Department of Radiation Oncology, Hospital Universitario Cruces/ Biocruces Health Research Institute, Plaza Cruces 12, 48903 Barakaldo, Spain; Department of Radiation Oncology, Instituto Catalan de Oncología, Avinguda de la Gran via de lʼHospitalet, 199-203, 08907 LʼHospitalet de Llobregat, Barcelona, Spain; Department of Radiation Oncology, Hospital de Castellón, Carrer de les Useres, 1, 12006 Castelló de la Plana, Castelló Spain; Department of Radiation Oncology, Hospital Meixoeiro, Meixoeiro, s/n, 36200 Vigo, Pontevedra, Spain; Department of Radiation Oncology, Hospital Virgen Del Rocío, Av Manuel Siurot, s/n, 41013 Sevilla, Spain; Department of Radiation Oncology, Hospital Virgen Macarena, Avd. Dr Fedriani, 3, 41071 Sevilla, Spain; Department of Radiation Oncology, Hospital San Chinarro, C/ Oña, 10, 28050 Madrid, Spain; Clinical Epidemiology Unit, Hospital Universitario Cruces/ Biocruces Heatlh Research Institute, Plaza Cruces 12, 48903 Barakaldo, Spain

**Keywords:** Pain flare, Palliative radiotherapy, Bone metastases

## Abstract

**Background:**

Palliative radiotherapy (RT) is an effective treatment for symptomatic bone metastases. Pain flare, a transient worsening of the bone pain after RT, has been described in previous reports with different incidence rates. The aim of the study was to prospectively evaluate the incidence of pain flare following RT for painful bone metastases and evaluate its effects on pain control and functionality of the patients.

**Methods:**

Between June 2010 and June 2014, 204 patients were enrolled in this study and 135 patients with complete data were evaluable. Pain flare was defined as a 2- point increase in worst pain score as compared with baseline with no decrease in analgesic intake or a 25 % increase in analgesic intake as compared with baseline with no decrease in worst pain score. All pain medications and worst pain scores were collected before, daily during, and for 10 days after RT. The Brief Pain Inventory (BPI) was filled out on the pretreatment and at the 4 weeks follow-up visit.

**Results:**

There were 90 men (66.7 %) and 45 women (33.3 %). Mean age was 66 years (SD 9.8). The most common primary cancer site was lung in 42 patients (31.1 %), followed by prostate in 27 patients (20.0 %). Forty-two patients (31.1 %) patients received a single fraction of 8 Gy and 83 (61.5 %) received 20 Gy in five fractions.

The overall pain flare incidence across all centers was 51/135 (37.7 %). The majority of pain flares occurred on days 1–5 (88.2 %). The mean duration of the pain flare was 3 days (SD: 3). There were no significant relationships between the occurrence of pain flare and collected variables.

All BPI items measured four weeks after end of RT showed significant improvement as compared with pretreatment scores (p < 0.001). No significant differences in BPI time trends were found between patients with and without flare pain.

**Conclusion:**

Pain flare is a common event, occurring in nearly 40 % of the patients that receive palliative RT for symptomatic bone metastases. This phenomenon is not a predictor for pain response.

## Background

Bone metastases are a common distance manifestation in advanced oncologic patients with a rising incidence due to longer survival of cancer patients. It is the most common cause of cancer-related pain and the most frequent symptom that requires treatment in cancer patients [1].

Palliative radiotherapy (RT) is a well-established and effective treatment alternative for symptomatic bone metastases. Depending on the criteria used, complete response of the pain could be achieved from 10 % to 35 % of patients, with overall pain response rates approaching 70 % [2].

Several randomized controlled trials (RCT) [3–7] and three meta-analysis [8–10] have shown equivalent pain response rates for single- and multiple-fraction radiotherapy treatments in the palliation of painful bone metastases.

Although RT for bone metastases is associated with limited side effects, a transitory aggravation of bone pain after treatment in the irradiated site has been recognized in several published reports, with published incidence rates of this phenomenon varying between 2 % and 40 % [11–13].

To date, the evidence in the literature evaluating prospectively the incidence of pain flare is scarce. Few prospective observational studies, with a low number of patients, have explored the incidence of pain flare [12, 14]. These studies have also demonstrated that pain flare associated with RT negatively impacts on the functionality and the mood of the patients.

Therefore, the objectives of this prospective multi-center observational study were to determine the incidence of pain flare after palliative radiation for painful bone metastases and to evaluate the impact of the pain flare on the pain control and the functionality of the patients.

## Methods and Materials

Subjects eligible for the study were patients with bone metastases treated with External Beam RT. Criteria for patient eligibility were: age 18 years or older with radiological evidence of bone metastases from a solid tumor, single lesion or multiple metastases were both accepted, pain intensity attributable to the bone lesion measured by Visual Analogue Scale (VAS) of 0–10 and patients capability and willingness to sign informed consent and complete the daily diary.

The radiation dose schedules permitted in the study were those administering 8 to 20 Gy in 1 to 5 daily fractions.

The protocol was initially approved by the Ethics Committee of the coordinating center (Hospital Universitario Cruces ), and ethics approval was subsequently requested and obtained at each participating center (Hospital Ramón y Cajal, Hospital Meixoeiro, Hospital Universitario Virgen de la Macarena, Hospital Gregorio Marañon, ICO- Instituto Catalan de Oncologia, Hospital Universitario Reina Sofia, Hospital Provincial Castellon, Hospital Sanchinarro, Hospital Virgen del Rocio).

### Patient evaluation

The pre-treatment evaluation consisted of a full history and physical examination, administration of Brief Pain Inventory (BPI), record of analgesic consumption within the previous 24 h and provision of the patient´s diary with oral and written instructions on how to fill it on a daily basis during treatment and 10 days after termination of RT. This diary recorded worst pain scores (on a numerical rating scale from 0–10), analgesic usage (total intake of analgesic medication in the 24 hour-period) and pain perception as compared with baseline pain (worse, better or same).

A follow-up visit was scheduled 4-weeks after the end of the RT. At this time the BPI was again administered, and analgesic consumption within the previous 24 hours was recorded.

### Treatment response

Pain response was measured using the International Bone Metastases Consensus from 2002 [15]. Complete response was defined as a pain score of zero at the bony metastatic site with no concomitant increase in analgesic intake. Partial response was defined as any of the following: a) pain reduction of two or more points below baseline score, at the bony metastatic site on a 0–10 scale without analgesic increase or b) analgesic reduction of 25 % or more from baseline without an increase in pain with reference to baseline. Pain progression was defined as an increase of pain score of two or more points above baseline at the bony metastatic site with stable analgesic use or an increase of 25 % or more daily OMED compared with baseline with pain score stable or one point above baseline. Patients not meeting these criteria were classified as having stable disease.

### Pain flare evaluation

Since most previous studies concerning pain flare have used the definition by Chow [11], we also chose to use this definition in order to enable comparisons with other studies.

Pain flare was defined (a priori) as any two-point increase of the pain scale of 0–10 in the daily diary compared to baseline levels with no decrease in analgesic intake or a 25 % increase in analgesic intake employing daily OMED with no decrease in pain score. If the baseline pain was nine, pain flare was defined if the follow up pain score was 10 accompanied with a response of current pain perception worse than the baseline pain. If the baseline pain was 10, pain flare was defined if the follow up pain score was 10 with current pain perception as worse than the baseline pain. To distinguish pain flare from progression of pain, we required the pain score and analgesic intake to return back to baseline after the flare [11].

### Statistical analysis

Descriptive statistics were used for demographic, clinical and treatment characteristics.

Continuous variables are expressed as the mean and standard deviation or median and range for quantitative variables, depending on distributional characteristics, whereas categorical variables are presented as frequencies and percentages.

Associations between categorical variables were assessed using Chi square or Fisher exact tests depending upon expected cell frequencies.

To assess changes in BPI items scoring between follow-up and baseline visit, T-tests for paired samples were used. To compare changes of BPI scores over time between patients with and without pain flare, T-tests for independent samples were used. All statistical analyses were conducted using SPSS v22 for Windows (IBM Corp. Released 2013. IBM SPSS Statistics for Windows, Version 22.0. Armonk, NY: IBM Corp). Statistical significance threshold was set at 5 %.

## Results

Between June 2010 and June 2014, 204 patients from ten Radiation Oncology Departments in Spain participated in this prospective observational study. From this cohort, 135 patients (66.2 %) completed the daily diary, 127 patients (62.2 %) the 4-weeks follow-up visit and 118 patients (57.8 %) completed both, the daily diary and the follow-up visit. These patients are labeled as evaluable patients and are the subject of statistical analysis. Reasons for incomplete data are: 25 patients experienced a deterioration in their performance status, 21 patients did not complete the pain diary, there were 16 unknown reasons for incomplete data and 15 patients requested removal from the study.

The relevant demographic, clinical and treatment characteristics of evaluable and no evaluable patients are shown in Table 1.

**Table 1:** Main demographic, clinical and treatment characteristics.

		Evaluable Patients (*N* = 135)	No Evaluable Patients (*N* = 69)
Age (years)	Mean (SD)	66.6(12.4)	65.6(11.1)
Gender	Male	90 (66.7 %)	45(65.2 %)
	Female	45 (33.3 %)	24(34.8 %)
Primary cancer site	Lung	42 (31.1 %)	21(30.4 %)
	Prostate	27 (20.0 %)	10(14.5 %)
	Breast	19 (14.1 %)	5(7.3 %)
	Others	47 (34.8 %)	33(47.8 %)
Worst pain^a^	Median (range)	8 (0–10)	8 (0–10)
RT dose	5 x 400 cGy	83 (61.5 %)	40(58.0 %)
	1 x 800 cGy	42 (31.1 %)	20(29.0 %)
	Other	10 (7.4 %)	9(13.0 %)
RT site	Pelvis	57 (42.2 %)	21(32.8 %)
	Spine	39 (28.9 %)	25(39.1 %)
	Extremities	20 (14.8 %)	11(17.2 %)
	Others	19 (14.1 %)	7(10.9 %)
Bisphosphonates	YES	36 (26.7 %)	16(23.2 %)
	NO	99 (73.3 %)	53(76.8 %)
Dexamethasone	YES	41 (30.8 %)	17(26.6 %)
	NO	92 (69.2 %)	47(73.4 %)

*Abbreviations*: RT = radiotherapy

^a^Worst pain recorded using the Brief Pain Inventory (BPI): ranged from 0–10

**Fig. 1:**
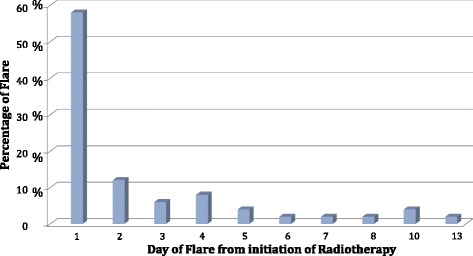
Distribution of pain flare events from the date of initiation of Radiotherapy

Based on the Chow definition [11], 51 of the 135 evaluable patients (37.7 %) presented pain flare. Pain flare occurred between days 1–5 after RT in 45/51 patients (88.2 %) and between days 6–15 in 6/51 patients (11.8 %) (Fig. 1). The mean duration of the flare was 3 days (SD 3).

Pain response to RT at 4 weeks was available for 127 of the 135 assessable cases. Fifteen patients (11.8 %) achieved a complete response and 64 (50.4 %) a partial response. Stable disease was found in 34 patients (26.8 %) and pain progression in 14 (11 %).

No significant relationships were found between the occurrence of pain flare and baseline variables, such as gender type, radiation dose, radiation site, primary cancer site, bisphosphonates use and dexamethasone use (Table 2).

**Table 2:** Relationships between the occurrence of pain flare and baseline variables (*n* = 135)

		FLARE		
		NO	YES	
		N (%)	N (%)	*p*-value*
Sex	Male	55 (61.1 %)	35 (38.9 %)	*p* = 0.706
	Female	29 (64.4 %)	16 (35.6 %)	
Cancer Site	Lung	23 (54.8 %)	19 (45.2 %)	*p* = 0.358
	Prostate	16 (59.3 %)	11 (40.7 %)	
	Breast	15 (78.9 %)	4 (21.1 %)	
	Others	30 (63.8 %)	17 (36.2 %)	
Bisphosphonates	YES	23 (63.9 %)	13 (36.1 %)	*p* = 0.810
	NO	61 (61.6 %)	38 (38.4 %)	
RT Dose	8 Gy/ 1 fx	28 (66.7 %)	14 (33.3 %)	*p* = 0.091
	20 Gy / 5 fx	47 (56.6 %)	36 (43.4 %)	
	Other	9 (90.0 %)	1 (10.0 %)	
Dexamethasone during RT	YES	25 (56.1 %)	18 (43.9 %)	*p* = 0.379
	NO	59 (64.1 %)	33 (35.9 %)	

*Abbreviations*: RT: radiotherapy; Fx: fraction

*p-value: chi-square/Fisher exact test

Changes in the BPI scores were assessed at 4 weeks after RT. For evaluation purposes, the BPI responses were assigned to two different dimensions, pain severity (sensory dimension of the pain) and pain interference (reactive dimension). There was a significant reduction in all BPI scores after RT (*p* < 0.001). A median decrease of 4 points compared to the pre-treatment setting was found for the “worst pain”, “activity”, “mood”, “walking ability” and “normal work” domains.

There were not significant differences in BPI changes between baseline and 4 weeks follow-up visits between patients with or without flare effect.

The BPI scores for those who responded (CR or PR) were compared with those who did not respond (SD or PD) to RT. There was a significant difference between the two groups in all functional interference items (*p* < 0.05) except for sleep (*p* = 0.549).

At 4 weeks, 70.8 % of evaluable patients with pain flare and 58.6 % of evaluable patients without flare were responders, respectively (p = 0.174).

## Discussion

This multisite prospective observational study shows that pain flare is a common event after palliative RT for painful bone metastases, affecting to almost 40 % of these patients. To our knowledge the current study is, up to the present time, the largest clinical cohort prospectively followed to determine the incidence and characterize the pain flare phenomenon. The use of a well-defined pain flare definition and the use of OMED values and verbal description of the pain in our investigation may have improved our capacity to identify a flare when compared with previous studies [13, 16].

Published articles have reported a wide range of the flare phenomenon, which vary between 2 % and 40 %. This difference could be at least partly explained by inconsistent definitions of pain flare, use of different radiation dose schedules, lack of adjustments for steroid medication or opiate medication, and variations in the time periods at which pain intensity was recorded to capture the flare event [17].

There are several reports [11, 12, 14, 17] specifically aimed at determining the incidence of pain flare (Table 3). Three of these studies used conventional 3DCRT and one of them stereotactic body radiation therapy (SBRT). While on the studies using 3DCRT the incidence was approximately 40 % [12, 14], the incidence in the study using SBRT was 68.3 %. This large difference in the incidence of pain flare has been postulated to be a result of significantly greater biologically effective doses delivered with SBRT [17].

**Table 3:** Published studies evaluating prospectively the incidence of Pain Flare

Author	Number of evaluable patients	RT technique	RT dose schedules (Gy/fx)	Pain Flare Incidence (%)
Chow	88	3DCRT	8 Gy / 1fx	2 – 16 %
			20 Gy 5 fx	
			30 Gy /10 fx	
Loblaw	44	3DCRT	8Gy / fx	40.9 %
			20 Gy/ 5 fx	
Hird	111	3DCRT	8 Gy / 1 fx	40 %
			20 Gy 5 fx	
			30 Gy /10 fx	
Chiang	41	SBRT	20-24 Gy / 1 fx	68.3 %
			24–35 Gy / 2–5 fx	
Gomez-Iturriaga	135	3DCRT	8Gy / fx	37.7 %
			20 Gy/ 5 fx	

Abbreviations: 3DCRT: Tridimensional Conformal Radiotherapy: SBRT: Stereotactic Body Radiotherapy

A study published by Pan et al. [18], found in a secondary analysis of single-institution phase 1/2 trials evaluating SBRT for the treatment of spinal metastases an overall incidence of flare after SBRT for spinal metastases of 23 %. A significant limitation of this study is that the trials were designed to investigate longer term symptomatic and tumor control outcomes instead of acute pain flare; hence, comparison between the studies is limited by differences in study design and population. Moreover, a recent study examining the Mayo Clinic experience of patients treated with SBRT to non-spine bony metastasis, found that only 10 % of the patients presented pain flare. Unfortunately, the flare definition used, the analgesic consumption pattern and the number of patients that received corticosteroids are unknown [19].

The pain flare incidence observed in the studies mentioned is higher than might be expected from clinical experience. In the study published by Chow et al. [11], there was an occurrence of 2 %-16 %, but subsequent reports by Loblaw et al. [12] and by Hird et al. [14] demonstrated pain flare rates of 40 %. This is comparable to what we have found in our study.

Based on our results apparently the advent of the pain flare is more common in the first days after the initiation of RT. Indeed, the flare occurred between days 1–5 after RT in 45/51 patients (88.2 %) and between days 6–15 in 6/51 patients (11.8 %). Hird et al. [14] reported similar results with 80 % of the patients presenting the flare within days 1–5 after the RT.

The physiopathology of the pain flare is largely unknown. It has been suggested to arise through edema of the periostium of the irradiated bone resulting in nerve compression or the release of inflammatory cytokines [20]. Thus, dexamethasone has shown potential for preventing this event. Two phase II studies, reporting on the effect of dexamethasone on the incidence of pain flare have been published [21, 22]; both studies reported rates of 24 % and 22 % respectively. In addition, recently, a phase III trial from Egypt has been published investigating the role of dexamethasone to prevent the flare phenomenon. They reported significant differences between groups, 16.2 % incidence in the dexamethasone group versus 38 % in the group without steroids (p = 0.0033) [23]. By contrast, in our study we have not found differences in pain flare incidence in favor of the patients who received corticosteroids. In order to explain these divergent findings, we must first acknowledge that our observational study was not specifically designed to address this issue. Moreover, less than half of our population received dexamethasone during RT (Table 2).

There is an ongoing RCT comparing two different dose schedules of dexamethasone and placebo led by the University Medical Center Utrecht. With an accrual goal of 411 patients, this study will help elucidate whether dexamethasone is an effective option in the prevention of pain flare after palliative radiotherapy for bone metastases [24].

Regarding pain response to RT, we found similar response rates to those reported by a range of RCT evaluating response to RT with different dose schedules. Foro [16], Chow [2], Hartsell [25] and Van der Linden [26] reported complete response rates ranging between 11 % and 25 %, partial responses between 26 % and 52 %, and overall response rates between 46 % and 72 %. Despite the elevated incidence of flare our results demonstrate 12 % of complete responses and 50 % of partial responses.

A secondary objective of our study was to evaluate the impact of the palliative radiotherapy on the pain control and the functional ability of the patients. The BPI is a well-known instrument, and has been shown to be a reliable and valid tool in depicting pain severity and the extent to which pain from bone metastases interferes with patient functioning. In the present study, significant changes in all BPI scores were found over time, decreasing from baseline to week 4.

Nguyen et al. [27] found in a study evaluating pain response and interference using BPI at 1, 3 and 6 months, that mood was significantly improved in patients responding to RT and a trend in improvement was observed for general activity and normal work. Another study by Wu et al. [28], in 109 patients who completed BPI after palliative RT for painful bone lesions at 4 and 6 weeks, reported a reduction for all seven functional interference items, with greatest improvement in general activity.

In our study we have found a significant difference in all BPI items after RT (p < 0.001) with larger difference (4 points) for the “activity”, “mood”, “walking ability” and “normal work” domains.

Most importantly, these observed significant differences in BPI items after RT have real impact on quality of life (QOL) and function, and provide clinically meaningful data to guide clinical decision-making [29].

In the interpretation of this study some limitations are acknowledged. These include a small sample size of 204 patients recruited and a reduction of the effective sample for final analysis to 135 patients. Loss to follow-up is a common problem in patients with metastatic disease. Furthermore, the limited BPI follow-up of 4 weeks after radiation impedes evaluation of long-term outcomes.

Our study demonstrates that pain flare is a common event in patients that receive palliative RT for bone metastases. Despite its frequency, it does not seem to have detrimental effect on the degree of pain control at 4 weeks. Our study has shown high rates of pain response and more importantly, a significant improvement on the functional interference dimension. Further studies evaluating whether simple interventions, such as prophylactic dexamethasone or short-acting opioids contribute to the prevention or amelioration of the flare phenomenon are warranted.

## Conclusion

Pain flare is a common event, occurring in almost 40 % of the patients that receive palliative RT for symptomatic bone metastases. RT for symptomatic bone metastases is a very effective palliative treatment, in terms of pain control and functional interference, even if the patient experiences in the short-term a pain flare.

## Abbreviations

BPI: Brief Pain Inventory
CR: Complete response
OMED: Oral Morphine Equivalent Dose
PR: Partial response
PD: Pain progression
QOL: Quality of life
RT: Radiotherapy
RCT: Randomized Controlled Trials
SBRT: Stereotactic body radiation therapy
SD: Stable disease
VAS: Visual Analogue Scale
3DCRT: 3D conformal radiotherapy
